# The Study of Release of Chlorhexidine from Preparations with Modified Thermosensitive Poly-*N*-isopropylacrylamide Microspheres

**DOI:** 10.1100/2012/243707

**Published:** 2012-04-26

**Authors:** Witold Musial, Bojana Voncina, Janusz Pluta, Vanja Kokol

**Affiliations:** ^1^Chair and Department of Pharmaceutical Technology, Wroclaw Medical University, ul. Szewska 38, 50-139 Wrocław, Poland; ^2^Department of Textile Materials and Design, University of Maribor, Smetanova ul. 17, 2000 Maribor, Slovenia

## Abstract

The aim of this study was to investigate and compare the release rates of chlorhexidine (CX) base entrapped in the polymeric beads of modified poly-*N*-isopropylacrylamides (pNIPAMs) at temperatures below and over the volume phase transition temperature (VPTT) of synthesized polymers: pNIPAM-A with terminal anionic groups resulting from potassium persulfate initiator, pNIPAM-B with cationic amidine terminal groups, and pNIPAM-C comprising anionic terminals, but with increased hydrophobicity maintained by the N-tert-butyl functional groups. The preparations, assessed in vitro below the VPTT, release an initial burst of CX at different time periods between 120 and 240 min, followed by a period of 24 h, when the rate of release remains approximately constant, approaching the zero-order kinetics; the release rates for the polymers beads are as follows: pNIPAM-C>pNIPAM-B>pNIPAM-A. The pattern of release rates at temperature over the VPTT is as follows: pNIPAM-C>pNIPAM-A>pNIPAM-B. In the presence of pNIPAM-C, the duration between the start of the release and the attained minimal inhibitory concentration (MIC) for most of the microbes, in conditions over the VPTT, increased from 60 to 90 min. The release prolongation could be ascribed to some interactions between the practically insoluble CX particle and the hydrophobic functional groups of the polymer.

## 1. Introduction

Chlorhexidine (CX), the known and widely applied antimicrobial agent, still is identified as the so-called “gold standard” in many applications, including oral and skin health applications, as well as some other skin and mucosal applications [1]. Researchers are evaluating numerous systems for controlled delivery of CX to the place of the interest, that is, mucosa of oral cavity, or skin surface. Yue et al. prepared, by single emulsion, solvent evaporation technique microparticles of poly(dL-lactic-co-glycolic acid) containing CX-free base, CX digluconate, and their association or inclusion complex with methylated-beta-cyclodextrin and hydroxypropyl-beta-cyclodextrin [2]. Some authors proposed urethane dimethacrylate-triethylene glycol dimethacrylate resin system with some success [3]. Early works included carriers based on acrylic strip [4] and chip of cross-linked collagen [5]. Also natural polymers as xanthan [6] or silk fibroin/gelatin hybrid films [7] were evaluated for loading CX.

The approach to effectively use microspheres for CX delivery to the place of proper activity was evaluated originally by Egbaria and Friedman [8], who evaluated the antibacterial activity of human albumin microspheres containing CX dihydrochloride against bacteria of urinary tract. The microspheres composed of chitosan were soon evaluated for preparation of buccal tablets with CX, acting on the oral mucosa [9]. Wu and Lee [10] applied in theirs microspheres loaded by chlorhexidin modified, that is, acetylated and succinoylated inulin-obtained microspheres were characterized by prolonged release of CX.

Poly-*N*-isopropylacrylamides (pNIPAM) are among the group of most applied macromolecules, characterized by reversible volume-phase transition at ca. 31°C [11]. The thermosensitive microgel particles may be applied in many medical devices, including drug forms for topical use [12, 13]. With the collapse and expansion of the macromolecule in the aqueous environment, the molecules of the biologically active substance may be released in a controlled manner [14, 15]. The deswelling process is controlled by diffusion, where the rate of the collapsing of the macromolecule is correlated to the dimensions of the pores in the polymeric matrix [16]. When the VPTT is crossed, the phase transition from one side enables the expelling of the drug from the polymeric environment, but on the other hand the aggregation may be expected, when the microspheres will adhere to the mucosa surface in the oral cavity.

In our previous studies, we investigated release of CX from ionic and nonionic polymer hydrogels, namely, from methylcellulose and modified polyacrylic acid [17]. Some approach was made to evaluate the amounts of CX released from modified poly-*N*-isopropylacrylamide by the conductometry method [18]. The aim of the present work is to evaluate the influence of three different microspheres batches on the release of CX in the conditions below and over the VPTT, applying pharmacopoeia release device.

The pNIPAM microgels, namely, pNIPAM-A, pNIPAM-B, and pNIPAM-C, with different terminal functional groups based on different initiators used in surfactant-free dispersion polymerization (SFDP), were synthesized in previous research [19]. For microgels pNIPAM-A and pNIPAM-C, potassium persulfate was used as an initiator in SFDP, while for pNIPAM-B, 2,2′-azo-bis(2-methylpropionamidine)dihydrochloride was used. The microgel pNIPAM-C was synthesized with the addition of comonomer, N-tert-butyl acrylamide, to increase the hydrophobicity of the received particles due to the experiments performed by Lynch et al. [20] as well as Lindmann et al. [21]. The IR spectra of the freeze-dried polymers obtained from SFEP were compared with that of the pure components to establish the occurrence of polymerization, and some physical assessments like optical microscopy, scanning electron microscopy, VPTT, conductivity, and pH were carried out to evaluate the products. The acquired release rates and concentrations of CX released from CX-loaded pNIPAM microgels were compared with that obtained from a water dispersion of CX as well as CX-loaded methylcellulose (MC) and polyacrylic acid (PA) beads [17].

## 2. Materials and Methods

N-isopropylacrylamide 97% (Aldrich), N-tert-butyl acrylamide 99% (Acros Organics), N,N′-methylenebisacrylamide 99% (Aldrich), potassium persulfate 98% (BDH Laboratory Suppliers (GPR), and 2,2′-azo-bis(2-methylpropionamidine) dihydrochloride 97% (Aldrich) were received from commercial and industrial suppliers and used without further purification. Dialysis bag of molecular weight cutoff (MWCO) of 12000–14000 Da was purchased from Visking Medicell International Ltd. Deionized water from the TKA DI 6000 system (Germany) was applied in all the procedures.

### 2.1. Synthesis of the Microgels

The N-isopropylacrylamide derivative microgel particles were synthesized by SFDP in deionized water at 70°C, under an inert nitrogen atmosphere due to the procedure reported in former paper [18]. The pNIPAM-A was characterized as a polymer with terminal anionic function groups. The pNIPAM-B was synthesized in the presence of 2,2′-azo-bis(2-methylpropionamidine)dihydrochloride, which resulted in cationic amidine terminal functional groups. The pNIPAM-C was characterized as the polymer with anionic terminal functional groups, but with increased hydrophobicity according to the functional groups introduced during the synthesis. For better evaluation of the polymerization process, the IR spectra assessments were performed to exclude the presence of vinyl groups in the products of the reaction, as well as to confirm the implementation of respective comonomer [18]. Also the other basic data on the characteristics of the microspheres were presented in the previous study, as the VPTT and scanning electron microscopy images of entities obtained through the synthesis.

### 2.2. Preparations of Microspheres-CX Mixtures

The CX-loaded preparations were developed using the polymers pNIPAM-A, pNIPAM-B, pNIPAM-C, and for comparison also MC and PA. The samples were allowed to swell in the 0.5% dispersions of CX for 48 h in a water bath at 298 K, under continuous stirring. After 48 h, when the incorporation procedure terminated, the samples were immediately frozen using liquid nitrogen and freeze-dried by MINI LYOTRAP LF/LYO/02/1 with vacuum pump model RV5, in high vacuum mode, at 50% power setting, with vacuum values in the range of 1 × 10^−1^–1 × 100 mbar (i.e., 1 × 10^1^–1 × 10^2^ Pa) for 24 h, and finally dispersed in water. The time for complete incorporation was demonstrated by previous consequent spectrophotometric assessments of the filtered samples, which came from the preparations loaded by chlorhexidine in various, increasing time periods. The exponential curve ranged the limit, and the amount of assessed drug did not decrease further after ca. 36 hrs. To receive the full load, the margin of 12 hrs was added. Composition of the obtained microgel preparations of CX is given in the attached Table 1.

### 2.3. Morphology of the Obtained Complexes of Polymer and CX

The surface and morphology of the freeze-dried samples were examined using light microscopy (LM). The morphology of the loaded samples was assessed by the optical microscope, Olympus BX51, with oculars 20 × 0.46 mm and 50 × 0.80 mm, and recorded using a digital camera, Olympus DP12, U-TVO.5XC-2, by applying direct day light. To obtain more data about the surface of the dry samples, the scanning electron microscope (SEM) was applied, using the FEI QUANTA 200 3D.

### 2.4. Release Rates

The in vitro release of CX from the solution in water and from the hydrogel preparations across artificial membrane was examined using United States Pharmacopoeia paddle method with the acceptor volume of 900 mL and a defined diffusional area of 64 cm^2^. Water was used as the acceptor phase, and it was adjusted to a pH of 5.5 by the addition of small quantities of 0.1 M HCl, to maintain the pH close to the physiological pH of the skin surface. After preparation, all the samples, that is, CX dispersion and loaded hydrogels, were placed in a thermostated water bath, at 22 and 37°C for 24 h. About 20 mL of the sample, either of the solution of CX or a hydrogel containing the drug, was placed in the donor compartment. Six experiments were conducted using these systems for a 24 h period. About 1 mL of receptor phase was taken as a sample and replaced with fresh prethermostated acceptor phase. The vessels were silanized using dichlorodimethyl silane to minimize CX adsorption on the glass. The samples were taken every 10 min for the first 100 min, then, every 30 min for the next 2 h, and, subsequently, every 1 h for up to 12 h, to achieve 20 measurement points for this time period. The CX in the eluent was assessed using the UV spectrophotometric method. The percentage extinction coefficient (a1%,1 cm) in 0.01 mol/L HCl solution was 224.12 (*P* > 0.999) at 250 nm, and the method gave a linear response over a concentration range of 1–20 *μ*g/mL [22]. The UV spectrophotometer, TECAN Infinite 200, with 96 wells plates, Greiner 96 flat-bottom transparent polystyrol plates, was used for the determination of CX concentrations in the studied samples.

## 3. Results and Discussion

The polymeric microgels obtained in the process of SFDP were prepared using known method proposed and evaluated, that is, by Pelton [23] as well as Saunders and Vincent [24]. Consequent IR assessments confirmed the reduction of the double bonds in the monomer molecule [25], demonstrating that the polymer was obtained [26]. The VPTT was observed in the turbidity measurements, and, hence, all the synthesized products were considered as thermosensitive [27]. In the present research, we confirmed the detailed evaluations performed earlier—the VPTT for pNIPAM-A and pNIPAM-B was around 34°C, whereas, for pNIPAM-C, it was observed at 32°. The conductivity measurements revealed complete purification of obtained material from the initiator or comonomer remains.

**Figure 1:**
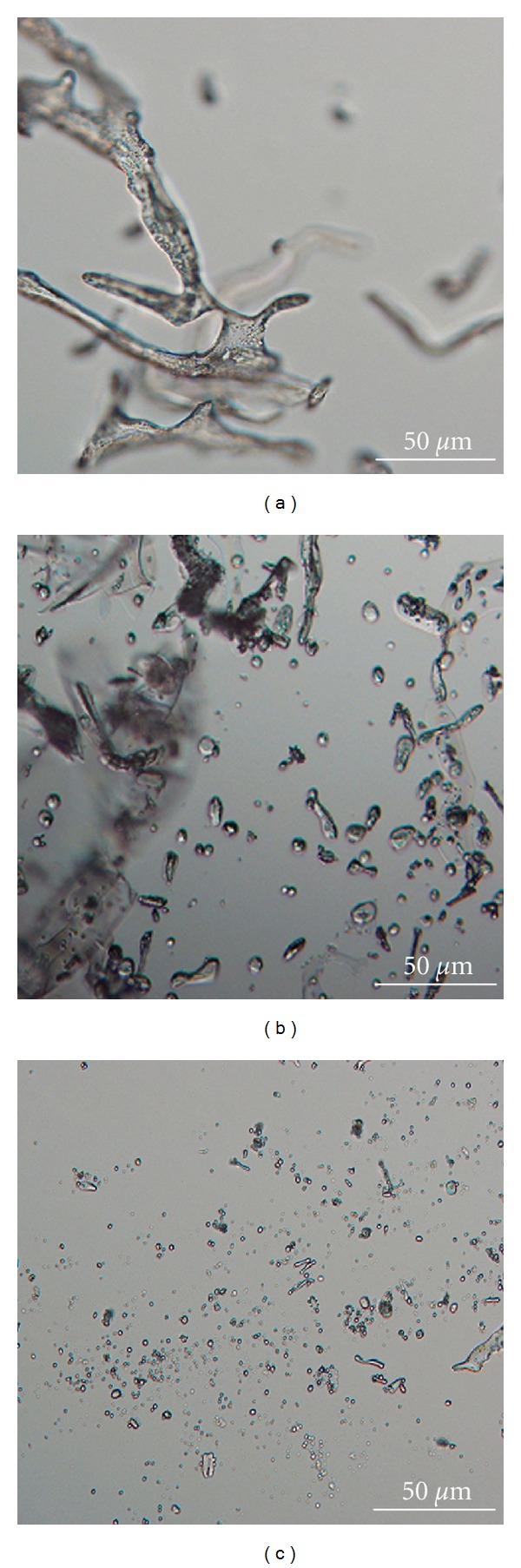
Morphology of obtained polymers; the light microscopy images: (a) pNIPAM-A, (b) pNIPAM-B, (c) pNIPAM-C.

**Figure 2:**
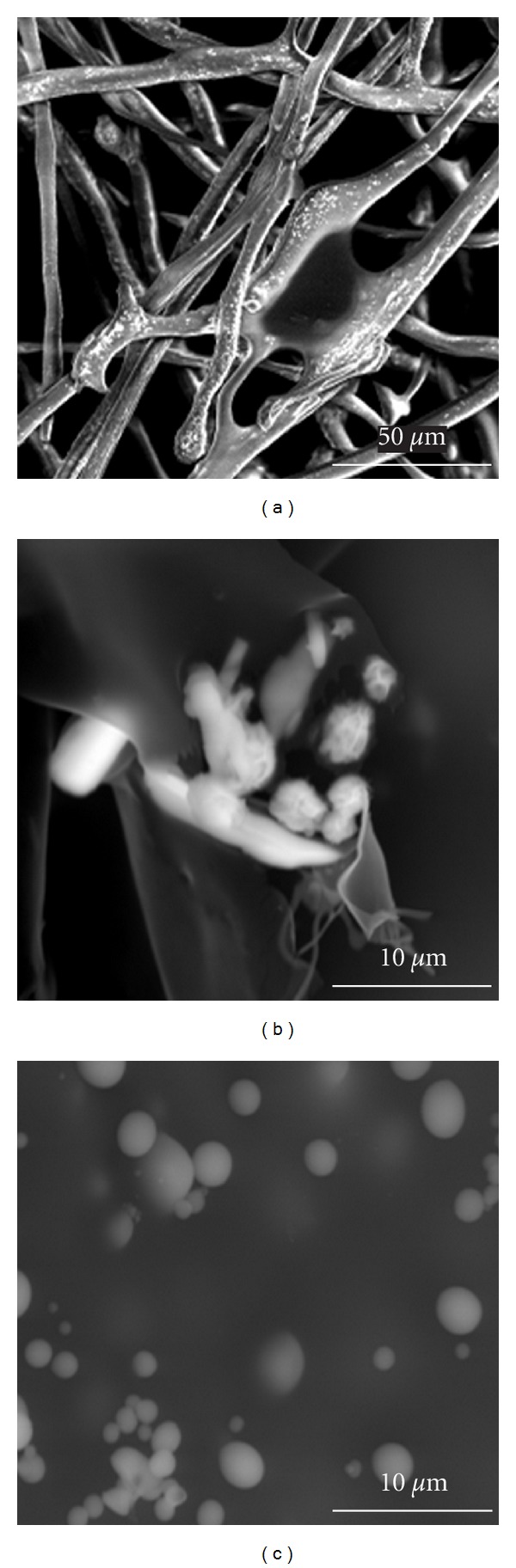
Morphology of obtained polymers; the scanning electron microscopy images: (a) pNIPAM-A, (b) pNIPAM-B, (c) pNIPAM-C.

Both LM and SEM observations of freeze-dried material revealed hydrogel-like structure in the case of CX-pNIPAM-A compositions, whereas for CX-pNIPAM-B and CX-pNIPAM-C compositions, we observed microspheres of diameter between 0.5 and 5 *μ*m (Figures 1 and 2). As it is evident from the LM microphotography, the spheres tended to aggregate, however, in the observation field, there were no particles actually aggregated. The SEM photograph, panel A (Figure 1), gives impression of some artifacts or deposited CX on the surface of the fiber-like structures. On the panel B of Figure 2, the obtained microgels have some observable empty areas, between planar surfaces of poly-*N*-isopropylacrylamide, very characteristic for this kind of material, possibly with some CX binded. This both interesting issues, dealing with suspected specific CX deposition on/in microgels, will be evaluated in next study. The hydrophobized particles had rather smooth surface in the SEM observations, with no observable artifacts, for example, CX crystals.

**Figure 3:**
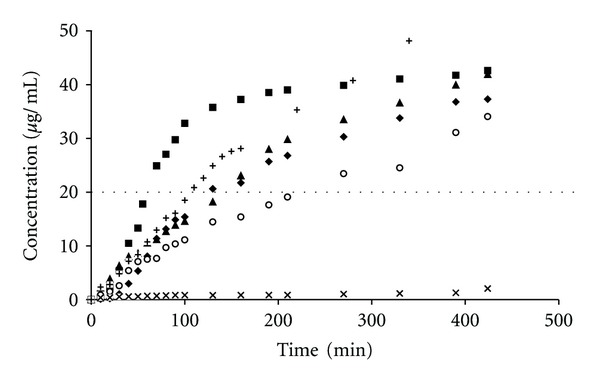
Influence of the type of polymer in the donor compartment on the CX concentration in the acceptor medium at 22°C. The release from respective preparations was depicted: pNIPAM-A-CX(♦), pNIPAM-B-CX (▲), pNIPAM-C-CX (■), MC-CX (∘), PA-CX (×), and aqueous dispersion of CX (+), data from repeated six experiments. The dashed straight line, parallel to the *x*-axis, represents the MIC of CX for *Staphylococcus mutans*.

**Figure 4:**
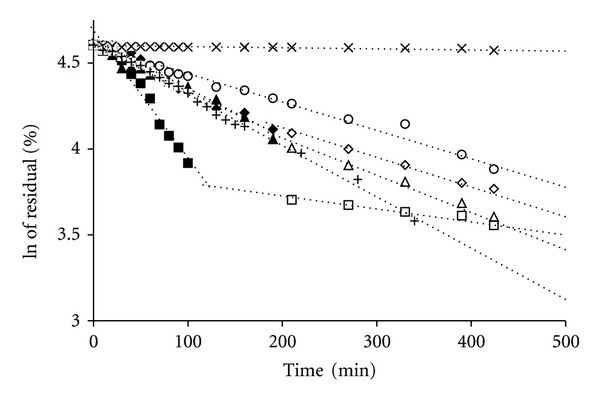
Influence of the type of polymer on the release kinetics of CX from the polymeric bead at 22°C. The release from respective preparations was depicted as follows: pNIPAM-A-CX for first stage (♦) and second stage (*◊*), pNIPAM-B-CX for first stage (▲) and second stage (∆), pNIPAM-C-CX for first stage (■) and second stage (□), MC-CX (∘), PA-CX (×), and aqueous dispersion of CX (+), data from repeated six experiments.

The CX preparations, assessed in vitro at 22°C, that is, below the VPTT for the studied microgels, released an initial burst of CX at different time periods between 120 and 240 min, followed by a period of ca. 24 h, in which the rate of release was approximately constant, approaching the zero-order kinetics as it is depicted in Figure 3. To determine whether the release rate decreased exponentially, the logarithms of the CX concentrations were plotted against time, which produced straight lines, as shown in Figure 4. The linear regression coefficients given in Table 2 were over 0.9584, indicating an extremely close fit of the values to the line and supporting the premise that the release rate decreased exponentially during the assay.

Depending on the preparations, the rates fell by approximately up to 10 times in a period of 24 h. During that period, the concentration of CX attained in each sample level of >20 *μ*g/mL, which is well above the minimal inhibitory concentration (MIC) for most of the microbes (Figure 3). The release rate was the highest in the case of aqueous suspension of the CX base. The release rates for the polymers beads were as follows: pNIPAM-C>pNIPAM-B>pNIPAM-A>MC>PA. Furthermore, the prolongation of CX release was ca. 30–50 min. The release of CX in the presence of pNIPAM can be divided into two stages (Table 3), and the transition point for pNIPAM-A and pNIPAM-B was observed at 90 min, whereas, for pNIPAM-C, the apparent release rate decreased significantly after 130 min. 

**Figure 5:**
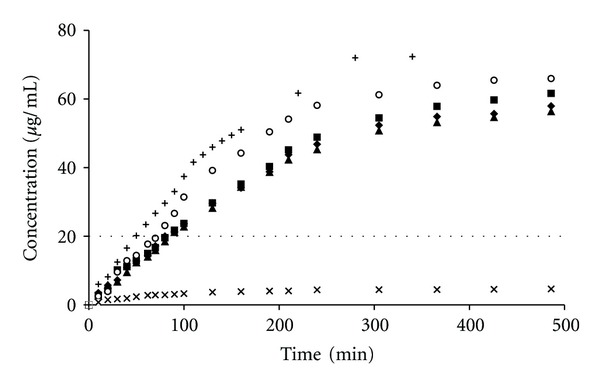
Influence of the type of polymer in the donor compartment on the CX concentration in the acceptor medium at 37°C. The release from respective preparations was depicted: pNIPAM-A-CX(♦), pNIPAM-B-CX (▲), pNIPAM-C-CX (■), MC-CX (∘), PA-CX (×), and aqueous dispersion of CX (+), data from repeated six experiments. The dashed straight line, parallel to the *x*-axis, represents the MIC of CX for *Staphylococcus mutans*.

**Figure 6:**
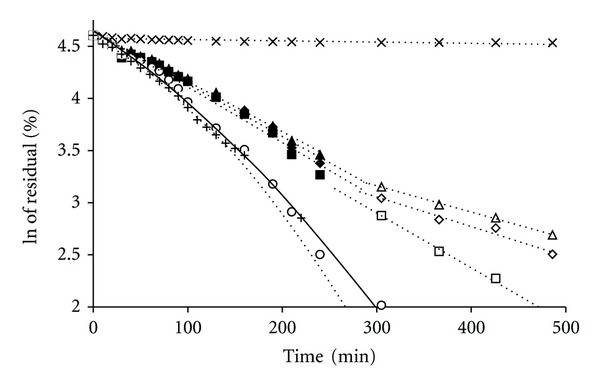
Influence of the type of polymer on the release kinetics of CX from the polymeric bead at 37°C. The release from respective preparations was depicted as follows: pNIPAM-A-CX for first stage (♦) and second stage (*◊*), pNIPAM-B-CX for first stage (▲) and second stage (∆), pNIPAM-C-CX for first stage (■) and second stage (□), MC-CX (∘), PA-CX (×), and aqueous dispersion of CX (+), data from repeated six experiments.

Additionally, the first-stage release rate for pNIPAM-C was significantly higher than those for pNIPAM-A and pNIPAM-B. Also, the release curves for pNIPAM-A and pNIPAM-B did not show much difference in group. For comparison, the respective data of the release rates for MC and PA are presented in Table 3, although the VPTT was not assessed for these polymers. For a specific time period, the release of CX at temperature over VPTT was different than that at temperature below VPTT (Figure 5). The acquired concentrations exceeded the known mean value of MIC of ca. 20 *μ*g/mL. Furthermore, the logarithms of the CX concentrations were plotted against time, as described earlier (Figure 6). The sequence of release rates for the assessed polymers can be presented as follows: MC>pNIPAM-C>pNIPAM-A>pNIPAM-B>PA. The highest release rate was observed for the aqueous suspension of the CX base. PA was the agent, which significantly influenced the release of CX. From the data presented in Table 3, it can be observed that the concentration of CX released from the PA preparation was extremely low.

To determine whether the synthesized polymers would influence the release kinetics of CX, an in vitro model was used, in which the samples of CX with polymer were exposed to a buffer under conditions recognized by the European Pharmacopoeia and the United States Pharmacopoeia as proper for the comparison of semisolid-drug forms applied topically. It would be unlikely for the release rates of CX and the concentrations observed in the acceptor fluid to be in exact agreement with those on the skin surface, where considerable variation in these parameters can be expected with regard to the differences in the location of the dermis, exposure to surface lipids, washing, and other effects observed on the skin surface. Nevertheless, the model system demonstrated that the characteristics of the kinetics of release differ among the applied polymers. Release systems, like those described in this study, in which a drug is dissolved or dispersed in a polymer vehicle, can be classified as diffusional matrix systems. They usually exhibit first-order release kinetics, in which the rate of release diminishes exponentially with time, in response to the decreasing concentrations of the drug in the polymeric matrix. However, the zero-order release in numerous cases is considered to be the ideal system for drug administration. That case is considered to occur within a matrix system, when the drug is present in the core of the matrix in a highly saturated state. Consequently, the diffusion of the drug from the matrix is rate limiting [28].

The data from the kinetics experiment were also employed for the evaluation of the maximum dose of CX that might be absorbed over an 8 h period by a patient receiving a topical treatment. The received concentration in the acceptor fluid was up to 13 mg/mL in the first hour, equivalent to a release of an estimated 1300 mg of the drug for the same period at a temperature over the VPTT, under skin conditions. This is far below the daily dose of 2000 mg of CX that humans are capable of ingesting without producing any adverse effects [29]. However, at temperature below VPTT, the values were not higher than 10 mg for pNIPAM-B and pNIPAM-C and reached almost 20 mg for pNIPAM-A. In this case, the evaluated concentration of CX on the skin reached the dose of 2000 mg. Thus, the differences in the release condition should be taken into account when thermosensitive particles are employed, such as drug carriers, for the topical and dermal applications as well.

The effective concentration of the drug on the skin surface was also evaluated in terms of the time period after which the concentration of MIC could be acquired. In general, the presence of polymers resulted in the decrease of release rate, with the extreme case of PA. When the temperature increased, the MIC in the acceptor fluid was observed in advance, and the MC preparation was the most significant case. On the other hand, the increase in the release rate and the corresponding decrease in the effective time can be elucidated in terms of the decrease in viscosity with respect to the increase in temperature. Nevertheless, in the presence of pNIPAM-C microgel, the effective time, that is, the duration since the start of the release to the moment when the MIC is reached, in thermal conditions over the VPTT, increased from 60 to 90 min. The pNIPAM-C polymer was characterized as a hydrophobized macromolecule, with butyl acrylate functional group—thus, the release prolongation could be attributed to some interactions between the insoluble CX molecule and the hydrophobic functional groups of the polymer. The assessed effective times are presented in Table 4.

The MIC of CX for *Streptococcus specia*, one of the main pathogens in the respiratory tract, is in the range of 0.25–64 *μ*g/mL, that is, 0.25–64 mg/L [30–32]. The proposed polymer systems for the release of active substance enable the achievement of direct bactericidal and bacteriostatic levels of CX in the application area after ca. 60–130 min, in the case of pNIPAM polymers at temperature below the VPTT. However, when the temperature is over the VPTT value for the assessed synthesized polymers, the antibacterial effect is observed at 80–90 min. The release of the CX from the polymeric preparations is observed to intensify simultaneously with the increase in the temperature during the release process. However, the only exception was pNIPAM-C. In the presence of this polymer, the effective time, that is, time needed to observe the MIC in the acceptor compartment, increased at the temperature over the VPTT. This can be owing to the effective embedding of the CX in the microsphere structure—one of the factors may be the lipophilic interaction of the polymer tertiary-butyl chains with the CX molecules. The future development of the N-isopropyl acrylamide microgels as drug carriers is supported by reported cytocompatibility of the pNIPAM nanoparticles [33]. The obtained data may be valuable for the development of new drug forms for controlled delivery of active substances onto the skin at different environmental temperatures, where the skin surface temperature could vary in the range from 14°C up to 42°C. Therefore, the therapeutic activity of the locally applied drug depends on both the thermodynamic activity of the active molecule compound and the pharmaceutical system, which enables the release of the molecule, as well as on the vasoconstrictive activity of the skin blood vessels. However, the release of the drug might be different when a patient is suffering from fever, when compared with the healthy subject. Furthermore, cryotherapy might also influence the release of the drug applied onto the skin.

## 4. Conclusions

The preparations evaluated in this study could be employed as CX carriers to achieve effective drug concentrations at different skin surface temperatures. The proposed polymer systems for the release of active substance enable the achievement of direct bactericidal and bacteriostatic levels of CX in the application area after ca. 60–130 min, in the case of pNIPAM polymers at temperature below the VPTT. The antibacterial effect is observed at 80–90 min when the temperature is over the VPTT value. Also, the data obtained for the in vitro preliminary selection of the thermosensitive polymers could be used for the further in vivo assays. This class of thermosensitive polymers can be further developed to achieve controlled release of the drug at different thermal conditions of the body.

## Figures and Tables

**Table 1:** Composition of investigated preparations.

Components preparation	CX (mg)	pNIPAM-A (mg)	pNIPAM-B (mg)	pNIPAM-C (mg)	MC (mg)	PA (mg)	Water (g)
pNIPAM-A-CX	45	90	—	—	—	—	20
pNIPAM-B-CX	45	—	90	—	—	—	20
pNIPAM-C-CX	45	—	—	90	—	—	20
MC-CX	45	—	—	—	90	—	20
PA-CX	45	—	—	—	—	90	20
H_2_O-CX	45	—	—	—	—	—	20

pNIPAM-A-CX, pNIPAM-B-CX, pNIPAM-C-CX: preparations of respective polymers detailed in the text with chlorhexidine, MC-CX: preparation of methylcellulose and chlorhexidine, PA-CX: preparation of polyacrylic acid and chlorhexidine, and H_2_O-CX: aqueous dispersion of chlorhexidine.

**Table 2:** Linear regression coefficients for the assessed release of CX from the polymeric preparations, assuming first-order process.

Release rates	Release rates assessed at 22°C (min^−1^)		Release rates assessed at 37°C (min^−1^)	
Preparation	1st stage	2nd stage	1st stage	2nd stage
pNIPAM-A-CX	0.9737	0.9864	0.9897	0.9675
pNIPAM-B-CX	0.9780	0.9783	0.9941	0.9967
pNIPAM-C-CX	0.9670	0.9584	0.9823	0.9960
MC-CX	0.9884	—	0.9838	—
PA-CX	0.8585	—	0.6922	—
H_2_O-CX	0.9949	—	0.9761	—

pNIPAM-A-CX, pNIPAM-B-CX, pNIPAM-C-CX: preparations of respective polymers detailed in the text with chlorhexidine, MC-CX: preparation of methylcellulose and chlorhexidine, PA-CX: preparation of polyacrylic acid and chlorhexidine, and H_2_O-CX: aqueous dispersion of chlorhexidine, data from repeated six experiments.

**Table 3:** Release rates of CX from polymeric preparations.

Polymer	Release rates assessed at 22°C (min^−1^)				Release rates assessed at 37°C (min^−1^)			
	1st stage	SD	2nd stage	SD	1st stage	SD	2nd stage	SD
pNIPAM-A-CX	2.85 × 10^−3^	0.01 × 10^−3^	1.74 × 10^−3^	0.02 × 10^−3^	4.98 × 10^−3^	0.02 × 10^−3^	2.80 × 10^−3^	0.26 × 10^−3^
pNIPAM-B-CX	2.62 × 10^−3^	0.02 × 10^−3^	2.15 × 10^−3^	0.05 × 10^−3^	4.79 × 10^−3^	0.04 × 10^−3^	2.50 × 10^−3^	0.18 × 10^−3^
pNIPAM-C-CX	7.43 × 10^−3^	0.04 × 10^−3^	7.57 × 10^−4^	0.40 × 10^−4^	5.37 × 10^−3^	0.04 × 10^−3^	5.26 × 10^−3^	0.39 × 10^−3^
MC-CX	1.66 × 10^−3^	0.03 × 10^−3^	—	—	1.22 × 10^−2^	0.01 × 10^−2^	—	—
PA-CX	3.60 × 10^−5^	0.04 × 10^−5^	—	—	4.09 × 10^−5^	0.05 × 10^−5^	—	—
H_2_O-CX	3.29 × 10^−3^	0.07 × 10^−3^	—	—	1.06 × 10^−2^	0.03 × 10^−2^	—	—

pNIPAM-A-CX, pNIPAM-B-CX, pNIPAM-C-CX: preparations of respective polymers detailed in the text with chlorhexidine, MC-CX: preparation of methylcellulose and chlorhexidine, PA-CX: preparation of polyacrylic acid and chlorhexidine, and H_2_O-CX: aqueous dispersion of chlorhexidine, data from repeated six experiments.

**Table 4:** Assessed effective times*.

Preparation	Effective time at the temperature of 22°C (min)	Effective time at the temperature of 37°C (min)	The decrease of time at which the MIC is acquired with the increase in the temperature in the range of 15°C (min)
pNIPAM-A-CX	130	80	50
pNIPAM-B-CX	145	80	65
pNIPAM-C-CX	60	90	−30
MC	210	70	140
CX	110	50	60
PA	n/a	n/a	n/a

*Effective time was recognized as the period from the start of the release to the moment when MIC for CX was observed, n/a: not assessed.
